# Icariin Attenuates Synaptic and Cognitive Deficits in an A*β*_1–42_-Induced Rat Model of Alzheimer's Disease

**DOI:** 10.1155/2017/7464872

**Published:** 2017-09-19

**Authors:** Chenxia Sheng, Panpan Xu, Kexin Zhou, Dan Deng, Chunhu Zhang, Zhe Wang

**Affiliations:** ^1^Department of Integrated Traditional Chinese and Western Medicine, Xiangya Hospital, Central South University, Changsha, Hunan 410008, China; ^2^Department of Integrated Traditional Chinese and Western Medicine, The Second Xiangya Hospital, Central South University, Changsha, Hunan 410011, China

## Abstract

Icariin (ICA), a prenylated flavanol glycoside present in abundant quantities in* Epimedium sagittatum*, has shown promise in the treatment and prevention of Alzheimer's disease. Damage to synaptic plasticity induced by amyloid-beta-mediated neurotoxicity is considered a main pathological mechanism driving the learning and memory deficits present in patients with Alzheimer's disease. This study investigated the neuroprotective effects of icariin in an A*β*_1–42_-induced rat model of Alzheimer's disease. Our results showed that A*β*_1–42_ injection induced loss of learning and memory behaviour in the Morris water maze, which could be reversed with intragastric administration of ICA. Furthermore, ICA reversed decreases in PSD-95, BDNF, pTrkB, pAkt, and pCREB expressions and prevented deterioration of synaptic interface structure. These findings indicate that ICA may improve synaptic plasticity through the BDNF/TrkB/Akt pathway and provide further evidence for its clinical application to improve learning and memory in patients with Alzheimer's disease.

## 1. Introduction

Alzheimer's disease (AD), a progressive neurodegenerative disease and the main cause of dementia, is characterized by gradual memory loss and deterioration of higher cognitive function [1, 2]. The accumulation and deposition of amyloid-beta (A*β*) in the brain affects the morphology and function of synapses by disrupting synaptic signaling pathways, thereby destroying synapses [3]. These changes lead to the deficits in memory and behaviour [4] which are key to AD pathology. Despite substantial research into AD, effective therapeutics remain elusive [5, 6]. Consequently, there is an urgent need for therapeutic strategies to overcome this disease.

Icariin (ICA), a major flavonoid constituent found in the Chinese medicinal herb* Epimedium brevicornum*, exerts a variety of pharmacological activities and has shown promise in the treatment and prevention of Alzheimer's disease [7]. We previously showed that Naoling decoction (NLD), a traditional Chinese medicine in which the major ingredient is ICA, was effective against AD, dose-dependently increased dendritic spines in the CA1 region, and protected against neuronal loss [8]. Our previous research also showed that ICA increased cell viability and decreased apoptosis in A*β*_25–35_-treated PC12 cells in association with activation of the PI3K/Akt signaling pathway [9]. Additionally, recent studies have reported that ICA can improve spatial learning ability and memory in rat model of AD by reducing the production of insoluble A*β* fragments, inhibiting abnormal tau protein phosphorylation, and exerting anti-inflammatory effects [10–13]. However, the mechanisms driving the neuroprotective role of icariin remain largely unknown.

Recently, synaptic plasticity deficits have become increasingly recognized as a cause of memory impairment and as central players in the synapse deterioration characteristic of AD pathogenesis [14]. Brain-derived neurotrophic factor (BDNF), a member of the neurotrophin family, is thought to promote synaptic growth, synaptic transmission, and synaptic plasticity. Moreover, BDNF exhibits cognition-promoting and neurotrophic effects by binding with its receptor tyrosine receptor kinase B (TrkB) and activating cyclic AMP-dependent response element-binding protein (CREB) [15, 16]. CREB, the proposed “final common pathway” of neurotrophin synthesis, plays a pivotal role in neuronal plasticity and durable memory formation when activated by phosphorylation at Ser133. Akt, also known as protein kinase B, is a major upstream modulator of CREB in neurotrophin-dependent signaling pathways and plays a vital role in the maintenance of synaptic plasticity and synaptogenesis [17, 18]. Taken together, these results suggest that the BDNF/TrkB/Akt pathway plays a crucial role in synaptic plasticity and is important in AD pathology [19, 20]. Furthermore, oridonin, isolated from the traditional Chinese herb* Rabdosia rubescens*, was shown to prevent A*β*_1–42_-induced synaptic loss by activating the BDNF/TrkB/CREB signaling pathway in the hippocampi of AD mice [21].

Based on the above findings, we hypothesized that ICA could ameliorate AD-induced cognitive impairment by regulating synaptic plasticity deficits through targeting the BDNF/TrkB/Akt pathway. In the current study, we examined the effects of ICA on A*β*_1–42_-induced synaptic loss and investigated the molecular mechanisms involved in the protection of synapses.

## 2. Materials and Methods

### 2.1. Animals and Experimental Design

**Figure 1:**
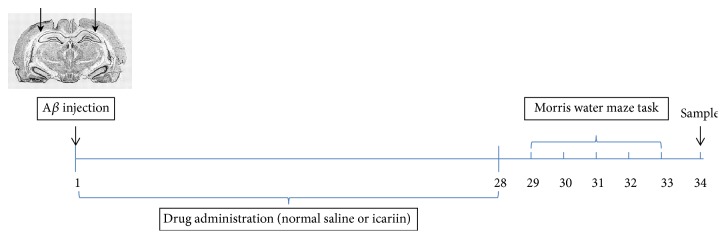
Design of experiments. Rats were microinjected with A*β*_1–42_ on day 1. They were given normal saline or icariin for 28 days, tested by the Morris water maze (MWM) for 5 days from the 29th day to the 33rd day, and sacrificed on day 34.

Sixty adult male Sprague-Dawley (SD) rats (weight: 200–250 g) were supplied by the Laboratory Animal Research Center of Central South University. The animals were housed under controlled conditions, including a 12-hour light/dark cycle, a temperature of 22–25°C, and 50 ± 10% relative humidity, with water and food pellets available ad libitum. All animals were given a 1-week acclimation period. The research was conducted in accordance with the ethical standards laid out in the Declaration of Helsinki as well as in the Guide for Care and Use of Laboratory Animals, adopted and promulgated by the United National Institutes of Health. The use of animals for experimental procedures was approved by the Review Committee of Central South University (Changsha, China). The authors' institutional review board approved this research. The total experimental period was 35 days (Figure 1). Rats were microinjected with A*β*_1–42_ on day 1. Rats were given normal saline or ICA for 28 days, tested by the Morris water maze (MWM) for 5 days from the 29th day to the 33rd day, and sacrificed on day 34.

### 2.2. Preparation of A*β*_1–42_ Solution

A*β*_1-42_ oligomer solution was prepared as described previously [22, 23]. Briefly, A*β*_1–42_ (Sigma, St. Louis, MO, USA) was dissolved in hexafluoroisopropanol (HFIP) at room temperature for 24 h. The HFIP was evaporated using a gentle stream of nitrogen gas, and the resulting peptide was dissolved in dimethyl sulfoxide (DMSO) at a final concentration of 1 mg/ml and stored at −20°C until use. The A*β*_1-42_ stock solution in DMSO was diluted directly by phosphate-buffered saline (PBS) to a final concentration of 2 *μ*g *μ*l^−1^, incubated at 4°C for 48 h, and used within 24 h.

### 2.3. A*β*_1–42_-Infused Rat Model

The A*β*_1–42_-infused rat model was established as described in our previous study [8]. After incubation, the aggregated A*β*_1–42_ peptides were injected into the intracerebroventricular (ICV) area of each animal to induce a validated AD model. The animals were anaesthetized with 10% chloral hydrate (4 ml/kg) and placed in a stereotactic frame. The A*β*_1–42_ oligomers (5 *μ*l*∗*2, Sigma, St. Louis, MO, USA) were injected bilaterally into the lateral ventricles through a microinjector with the following coordinates: 1.1 mm posterior to the bregma, 2.2 mm lateral to the sagittal suture, and 3.0 mm beneath the dura. For the sham group, an equivalent volume of PBS was injected bilaterally into the lateral ventricles through a microinjector using the above-mentioned coordinates.

### 2.4. Chemicals and Drug Treatment

ICA (purity above 98%) was purchased from the National Institute for the Control of Pharmaceutical and Biological Products (Beijing, China) and freshly dissolved into saline every day between 8 and 9 am to create 30, 60, and 120 mg/ml ICA solutions [24]. The rats were randomly distributed into five groups (*n* = 12 per group): (1) sham-operated (sham) group, including rats that underwent the above A*β*_1–42_-infusion procedure with bilateral ventricular injection of an equal volume of PBS without A*β*_1–42_ and were gavaged with the same amount of 0.9% NaCl; (2) A*β*_1–42_-infused + vehicle (vehicle) group, including rats that underwent A*β*_1–42_-infusion along with intragastric administration of an equal volume of 0.9% NaCl; (3) A*β*_1–42_-infused + 30 mg/kg ICA (ICA-L) group, including A*β*_1–42_-infused rats that were given ICA (30 mg/kg) via gavage; (4) A*β*_1–42_-infused + 60 mg/kg ICA (ICA-M) group, including A*β*_1–42_-infused rats that were given ICA (60 mg/kg) via gavage; and (5) A*β*_1–42_-infused + 120 mg/kg ICA (ICA-H) group, including A*β*_1–42_-infused rats that were given ICA (120 mg/kg) via gavage. The dosage regimen for each group was performed once per day after A*β*_1–42_ infusion for 28 days.

### 2.5. Morris Water Maze

The Morris water maze test was performed as previously described [25] 28 days after the start of the ICA intervention to evaluate spatial learning and memory deficits. The ANY-maze video tracking system (Stoelting Co., USA) was used to track and analyse behavioural parameters. The navigation test was performed once per day from the 29th day to the 33rd day after operation. The rats were introduced into the pool at one of four entry points, and every entry point was used over the course of a day. The rats were given 60 s to locate the platform and 10 s to remain on it once located. Rats unable to locate the platform in 60 s were placed on the platform for 10 s before being removed from the pool. The escape latency from being put into the water to finding and climbing onto the platform was observed and recorded. A spatial probe trial was performed to assess the strength of spatial memory retention on the 33rd day. In this test, the platform was removed, and the number of platform crossings, the run percentage in the target quadrant, and the time spent in the target quadrant were recorded over one 90 s trial.

### 2.6. Ultrastructure Detection

Transmission electron microscopy analysis of synapses was conducted according to a previous study [26, 27] after 28 days of the ICA intervention. Briefly, half of each rat's hippocampus was separated, fixed with 2.5% glutaraldehyde, and cut into pieces (100 *μ*m). The samples were fixed, dehydrated, soaked, and embedded through a graded acetone series. Finally, the embedded sections were dual-stained with uranyl acetate and lead citrate. The middle third of the CA1 stratum radiatum was observed with a transmission electron microscope (Hitachi Ltd., Tokyo, Japan) and shown on an image analyser (Hitachi Ltd., Tokyo, Japan). The number of synapses, the width of each synaptic cleft, the thickness of the postsynaptic density, the length of the synaptic active zone, the chord length, and the arc length of the postsynaptic membrane were measured. The percentages of flat synapses and perforated synapses were statistically analysed.

### 2.7. Immunohistochemical Analysis

Immunohistochemistry (IHC) was performed to visualize BDNF and pCREB as described in a previous study. Sections of each rat's hippocampus were boiled in 0.01 M citrate buffer (pH 6.0) for 10 min and then rinsed with phosphate-buffered saline (PBS). Endogenous peroxidase was blocked with methanol containing 0.3% hydrogen peroxide for 20 min. Serial sections were separately incubated overnight at 4°C with primary antibodies against either BDNF (1 : 500, Proteintech, USA) or pCREB (Ser133, 1 : 200, Santa Cruz, USA). After washing, the sections were incubated at room temperature with an anti-rabbit secondary antibody (1 : 10,000, Abcam, USA) for 1 h. At least six to ten alternate sections per rat hippocampus were imaged and analysed.

### 2.8. Western Blotting

Samples of the hippocampus were homogenized on ice (12,000 rpm, 4°C, 20 min) and quantified using a Bradford assay (BioRad, USA). Nuclear protein was obtained using NE-PER® Nuclear and Cytoplasmic Extraction Reagents (CER, Pierce Biotechnology, USA). The BCA method was used to measure total protein. In total, 50 *μ*g of protein lysate was separated by electrophoresis and transferred to a polyvinylidene difluoride membrane under semidry conditions at 250 mA.

After blocking in TBST buffer, the membranes were incubated with the following primary antibodies: PSD-95 (1 : 500, Proteintech, USA), BDNF (1 : 500, Proteintech, USA), pTrkB (Thr706, 1 : 400, Santa Cruz, USA), TrkB (1 : 1000, Santa Cruz, USA), pAkt (Ser473, 1 : 1000, Proteintech, USA), Akt (1 : 500, Proteintech, USA), pCREB (Ser133, 1 : 200, Santa Cruz, USA), and CREB (1 : 1000, Proteintech, USA). Then, the membranes were incubated with a horseradish peroxidase- (HRP-) conjugated secondary antibody (Abcam, USA). Protein signal intensities were detected with an ECL kit (Thermo) and visualized by exposure to Kodak film. The amount of protein was expressed as a relative value to the levels of *β*-actin.

### 2.9. Real-Time Quantitative RT-PCR

As described previously [25], quantitative real-time PCR (qPCR) was performed using 50 ng cDNA and custom-designed primers for BDNF, TrkB, Akt, and CREB. Quantitative real-time- (qRT-) PCR (Arraystar) was performed using an Applied Biosystems ViiA 7 Real-Time PCR System and 2x PCR Master Mix. Total RNA was isolated from hippocampal tissues and reverse-transcribed into cDNA using SuperScript III Reverse Transcriptase (Invitrogen, Grand Island, NY, USA). Primers were chemically synthesized (Sangon Biotech, Shanghai, CHN). The incubation conditions were 95°C for 10 min, followed by 40 cycles of 95°C for 10 s and 60°C for 1 min. Relative mRNA expression was calculated using the DDCT method and normalized to *β*-actin.

### 2.10. Statistical Analysis

Data from all procedures are expressed as the mean ± SD and were analysed with SPSS 21.0 (IBM, Armonk, NY, USA). Diagrams were plotted with Prism GraphPad software. Statistical analysis of escape latency was performed using repeated-measures ANOVA analysis, and all other data were subjected to one-way ANOVA followed by LSD comparison. *p* < 0.05 was considered as significant.

## 3. Results

### 3.1. ICA Improved Cognitive Recovery after Intracerebroventricular Injection of A*β*_1–42_

**Figure 2:**
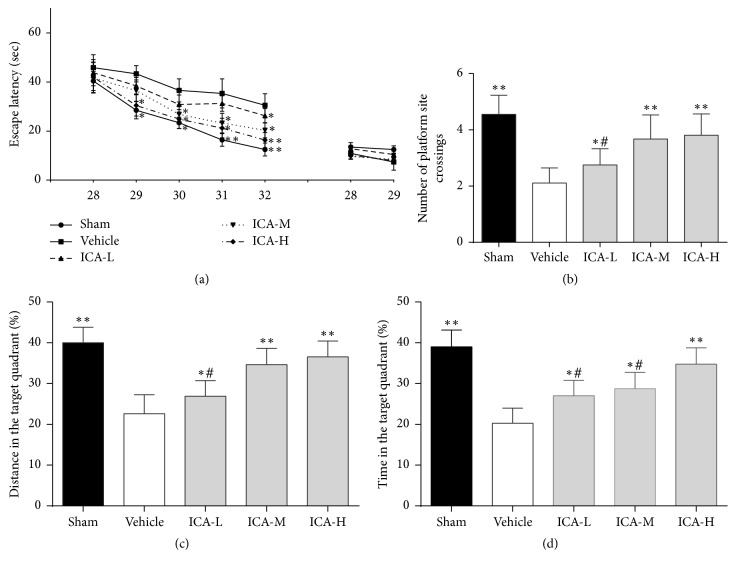
Effect of ICA on cognitive outcomes in A*β*_1–42_-infused rat model. Rats that received intracerebroventricular injection of A*β*_1–42_ were gavaged with ICA (ICA-L group: 30 mg/kg; ICA-M group: 60 mg/kg; ICA-H group: 120 mg/kg) or an equivalent amount of saline (vehicle group) each day. Sham-operated rats (sham group) also received an equal volume of saline orally. The Morris water maze test was then performed to evaluate cognitive function from the 28th day to the 32nd day. Significant group effects were observed in the hidden platform test used to assess the impact of ICA treatment, but no differences in visible platform performance were noted (a). The probe tests performed on the 32nd day showed significantly increased numbers of rats crossing the platform and (b) higher residence distance (c) and time (d) in the target quadrant for the ICA groups compared with the vehicle group. Escape latency data were analysed with two-way ANOVA, and probe test data were analysed with one-way ANOVA. All data are presented as the mean ± SD. ^*∗*^*p* < 0.05 and ^*∗∗*^*p* < 0.01 versus the vehicle group. ^#^*p* < 0.05 versus the ICA-H group.

The Morris water maze test was used to assess the effect of ICA on cognitive function in A*β*_1–42_-infused rats. As shown in Figure 2, the escape latency was clearly increased in the vehicle group compared with the sham group from the 29th day to the 32nd day (on the 29th day, *p* < 0.05; from the 30th day to the 32nd day, all *p* < 0.01). Treatment with ICA significantly reduced the increased escape latency compared with the vehicle group: ICA-L, only on the 32nd day, *p* < 0.05; ICA-M, from the 29th day to the 31st day, *p* < 0.05, and on the 32nd day, *p* < 0.01; ICA-H, from the 29th day to the 30th day, *p* < 0.05, and from the 31st day to 32nd day, *p* < 0.01. No significant differences were found in the visible platform test between groups, indicating that the impaired mobility from the injection of A*β*_1–42_ did not influence cognitive function. The probe tests performed on the 32nd day, the number of platform crossings (Figure 2(b)), the run percentage in the target quadrant (Figure 2(c)), and the time spent in the target quadrant (Figure 2(d)) were significantly decreased in the vehicle group compared to the sham group (*p* < 0.01). Furthermore, ICA treatment significantly increased the number of rats crossing the platform, the run percentage in the target quadrant, and the time spent in the target quadrant, which were decreased in the vehicle group, in a dose-dependent manner (Figures 2(b)–2(d)).

### 3.2. Effect of ICA on Synaptic Ultrastructure in the CA1 Region in A*β*_1–42_-Infused Rats

**Figure 3:**
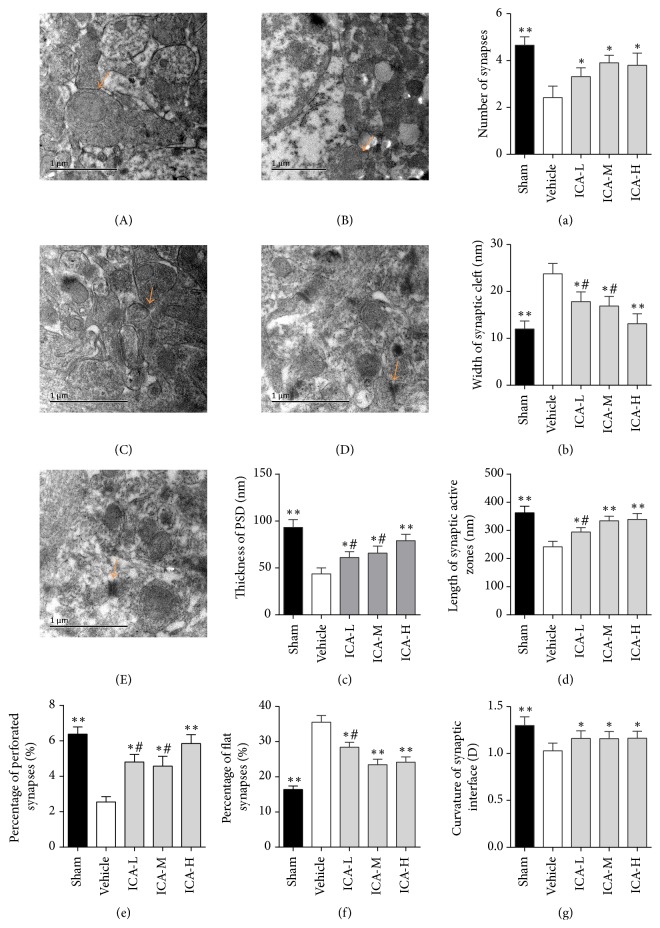
Effect of ICA on synaptic ultrastructure in the CA1 region of the hippocampi of A*β*_1–42_-infused rats (×10,000). Typical electron photomicrographs of different groups. (A) Sham group, (B) vehicle group, (C) ICA-L group, (D) ICA-M group, and (E) ICA-H group. Statistical graphs showing the parameters of synaptic structure (a–g) in the CA1 region. (a) Number of synapses. (b) Width of synaptic cleft. (c) Thickness of PSD. (d) Length of synaptic active zone. (e) Percentage of perforated synapses. (f) Percentage of flat synapses. (g) Curvature of synaptic interface. Sham group: normal presynaptic mitochondria, postsynaptic densities, synaptic cleft, and concave synapses. Vehicle group: unclear presynaptic mitochondria, thin postsynaptic density, reduced concave synapses, increased synaptic cleft, and flat synapses. ICA treatment group: compared with the vehicle group, the synaptic cleft is narrowed, the synaptic active zones are longer, the postsynaptic density is thicker, and the synapses have become curved. All data were analysed using one-way ANOVA and are presented as the mean ± SD. ^*∗*^*p* < 0.05 and ^*∗∗*^*p* < 0.01 versus the vehicle group. ^#^*p* < 0.05 versus the ICA-H group.

Compared with the sham group, there were significantly fewer synapses, an increased synaptic cleft width, thinner postsynaptic densities, shorter synaptic active zones, decreased synaptic cleft curvature, significantly fewer perforated synapses, and significantly more flat synapses in the vehicle group (all *p* < 0.01, Figure 3). Compared with the vehicle group, the ICA-treated rats showed an increased number of synapses, decreased synaptic cleft width, increased PSD thickness, longer synaptic active zone, increased proportion of perforated synapses, decreased proportion of flat synapses, and increased synaptic cleft curvature. It is notable that the ICA-H group showed better synaptic cleft width and postsynaptic density thickness than the ICA-L group and ICA-M group. The ICA-H group showed better synaptic active zone length, percentage of flat synapses, and percentage of perforated synapses than the ICA-L group. These results indicate that ICA rescued the synapse failure induced by A*β* deposition in AD rats with a positive dose-effect relationship.

### 3.3. Effect of ICA on BDNF and pCREB Expression in Immunoreactive Cells in the Hippocampi of A*β*_1–42_-Infused Rats

**Figure 4:**
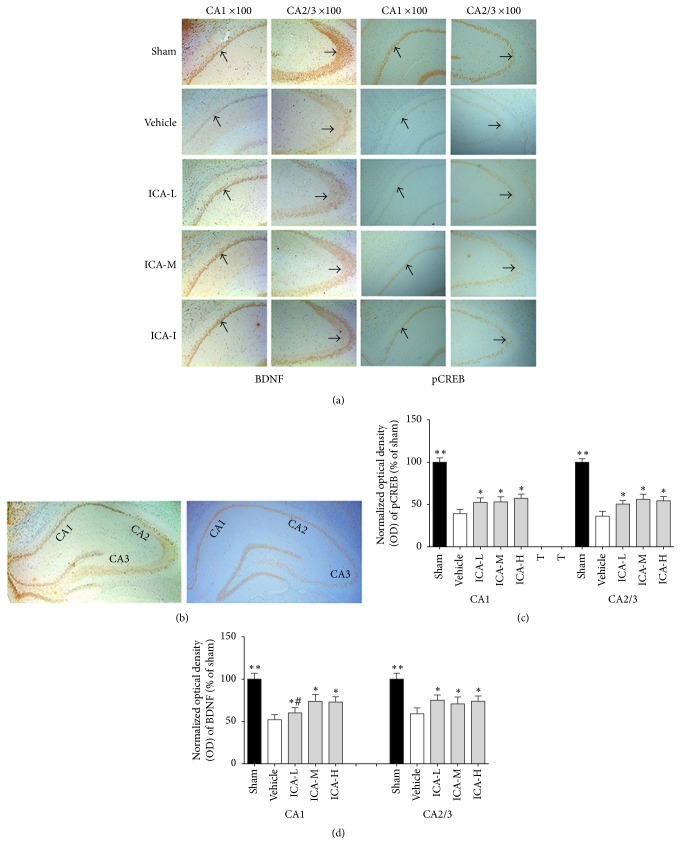
Effect of ICA on the expression of BDNF and pCREB in immunoreactive cells in the hippocampi of A*β*_1–42_-infused rats. (a) Representative immunohistochemistry images of BDNF and pCREB expression in the sham, vehicle, ICA-L, ICA-M, and ICA-H groups (*n* = 5/group). The selected CA1 regions are shown in the first (BDNF) and third (pCREB) columns, and the selected CA2/3 regions are shown in the second (BDNF) and forth (pCREB) columns (amplification ×100). The arrows indicate immunopositive cells. (b) Representative immunohistochemistry images of BDNF (left) and pCREB (right) in the hippocampus (amplification ×40). (c) Statistical graph showing the OD of BDNF. (d) Statistical graph showing the OD of pCREB. The results are expressed as the percentage relative to the sham group. All data were analysed using one-way ANOVA and are presented as the mean ± SD. ^*∗*^*p* < 0.05 and ^*∗∗*^*p* < 0.01 versus the vehicle group. ^#^*p* < 0.05 versus the ICA-H group.

The average numbers of BDNF- and pCREB-immunopositive cells were measured after 28 days of ICA or normal saline treatment in all five groups. As shown in Figure 4, there were significantly fewer BDNF- and pCREB-immunopositive cells in the vehicle group compared with the sham group. ICA treatment significantly increased the proportion of BDNF- and pCREB-immunopositive cells at a low dose, medium dose, and high dose (*p* < 0.05, *p* < 0.01, and *p* < 0.01, resp.) in the CA1 and C2/3 regions of the hippocampus (Figures 4(a)–4(d)).

### 3.4. Effect of ICA on PSD-95, BDNF, pTrkB, pAkt, and pCREB Protein Expressions in the Hippocampi of A*β*_1–42_-Infused Rats

**Figure 5:**
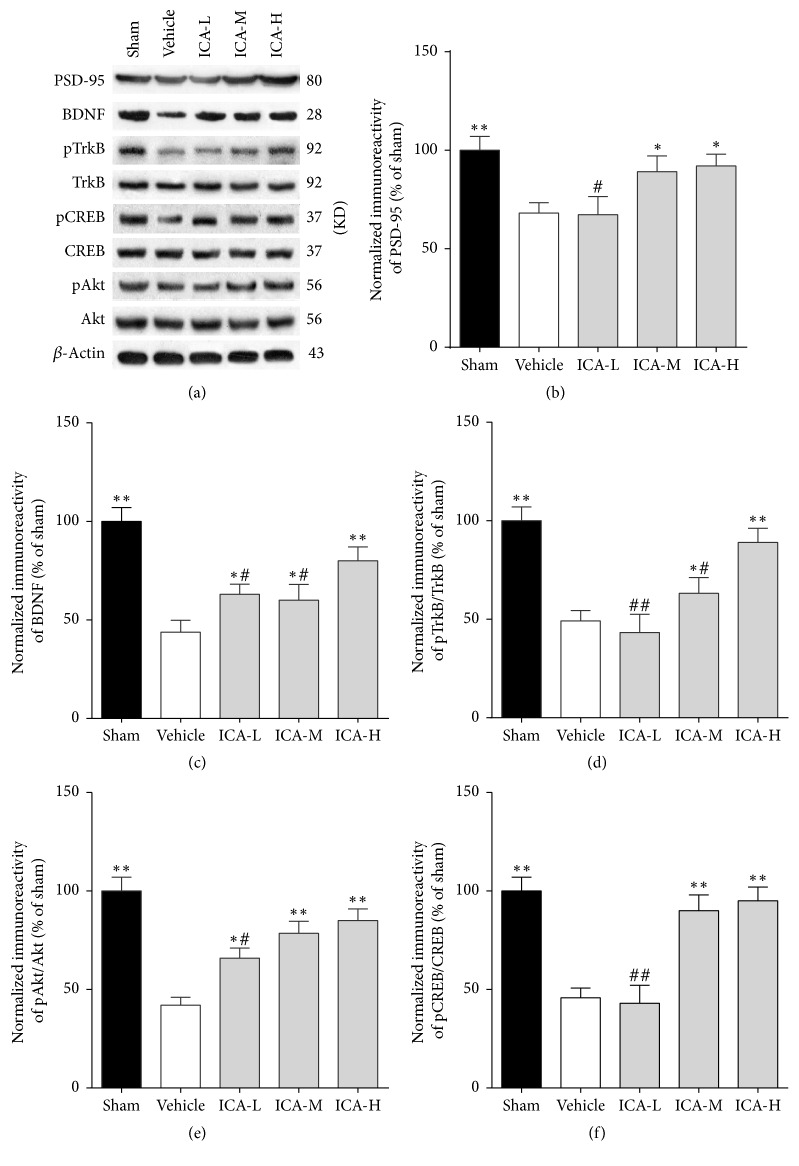
Effect of ICA on PSD-95, BDNF, pTrkB, pCREB, and pAkt protein expressions in the hippocampi of A*β*_1–42_-infused rats. (a) Representative Western blot images showing PSD-95, BDNF, pTrkB/TrkB, pAkt/Akt, and pCREB/CREB expressions in the hippocampi of different groups. *β*-Actin was included as a loading control. (b, c, d, e, and f) PSD-95, BDNF, pTrkB/TrkB, pAkt/Akt, and pCREB/CREB expressions. The results are expressed as a percentage of the sham group. All data were analysed using one-way ANOVA and are presented as the mean ± SD. ^*∗*^*p* < 0.05 and ^*∗∗*^*p* < 0.01 versus the vehicle group. ^#^*p* < 0.05 and ^##^*p* < 0.01 versus the ICA-H group.

Western blot analysis of hippocampal samples was performed on day 34. As shown in Figure 5, the expressions of PSD-95, BDNF, pTrkB, pAkt, and pCREB in the model group were significantly lower than those in the sham group (*p* < 0.01). Compared to the vehicle group, ICA treatment significantly increased the protein expression of PSD-95 at medium and high doses (*p* < 0.05), and BDNF produced the same effect at low, medium, and high doses (*p* < 0.05, *p* < 0.05, and *p* < 0.01, resp.). In contrast, TrkB, Akt, and CREB protein expressions did not significantly differ among the five groups; however, ICA treatment significantly increased the pTrkB/TrkB ratio at low, medium, and high doses (*p* < 0.05, *p* < 0.05, and *p* < 0.01, resp.). pAkt/Akt produced the same effect at low, medium, and high doses (*p* < 0.05, *p* < 0.01, and *p* < 0.01, resp.), and pCREB/CREB showed similar results at medium and high doses (*p* < 0.01). These findings indicate that activation of the BDNF/TrkB/Akt pathway might be responsible for the neuroprotection induced by ICA.

### 3.5. Effect of ICA on mRNA Expression of BDNF, TrkB, CREB, and Akt in the Hippocampi of A*β*_1–42_-Infused Rats

**Figure 6:**
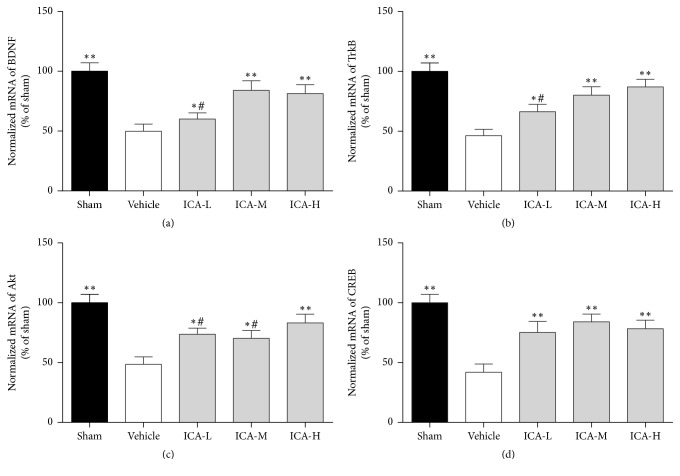
Effect of ICA on mRNA expression of BDNF, TrkB, CREB, and Akt in the hippocampi of A*β*_1–42_-infused rats. Statistical graph showing quantification of mRNA expression of BDNF (a), TrkB (b), Akt (c), and CREB (d). The results are expressed as a percentage of the sham group. All data were analysed with one-way ANOVA and are presented as the mean ± SD. ^*∗*^*p* < 0.05 and ^*∗∗*^*p* < 0.01 versus the vehicle group. ^#^*p* < 0.05 versus the ICA-H group.

BDNF, TrkB, Akt, and CREB mRNA expressions were measured via qRT-PCR. Compared to the sham group, the group that underwent A*β*_1–42_ injection into the paracele showed significantly decreased BDNF, TrkB, Akt, and CREB mRNA expressions in the hippocampus (all *p* < 0.01, Figure 6). ICA treatment significantly rescued the reduced mRNA levels of BDNF (Figure 6(a)) and TrkB (Figure 6(b)) at low, medium, and high doses (*p* < 0.05, *p* < 0.01, and *p* < 0.01, resp.). Akt caused the same effect (Figure 6(c)) at low, medium, and high doses (*p* < 0.05, *p* < 0.05, and *p* < 0.01, resp.) as did CREB (Figure 6(d)) (all *p* < 0.01).

## 4. Discussion

In this study, A*β*-treated rats showed deterioration in learning and memory in the Morris water maze test. Specifically, their escape latency time was prolonged, the time they spent in the target quadrant was reduced, and their synaptic structures were damaged. After the ICA intervention, their escape latency was shortened, their time spent in the target quadrant increased, their number of synapses increased, and their synaptic ultrastructure was restored. These changes were accompanied by upregulation of the synaptic protein PSD-95 in the hippocampus, as well as upregulation of BDNF, phosphorylated TrkB, Akt, and CREB. Collectively, these results show that ICA relieved the learning and memory deterioration and synaptic dysfunction caused by A*β* through the BDNF/TrkB/Akt pathway, inducing protective effects in the brain.

Emerging evidence has demonstrated that the pathological basis driving the cognitive alterations in AD is damage to synaptic plasticity and neuronal loss induced by early extracellular aggregation of A*β*. Based on these findings, new approaches targeting synapses could become effective disease-modifying therapeutics [28]. Brain tissues from AD patients show a marked loss of synapses, which could underlie the cognitive decline experienced by these individuals [29]. Synaptic structural plasticity is a key element in understanding various memory phenomena [30]. Damage to synaptic morphology and structure in the hippocampus correlates with the severity of the neuropathology and memory deficits present in individuals with AD [31]. In this study, we showed that administration of ICA may improve learning and memory by altering synaptic morphology and structure. For example, the treatment increased the number of synapses, the percentage of perforated synapses, the synaptic active zone length, the PSD thickness, and the synaptic curvature while narrowing the synaptic cleft and reducing the percentage of flat synapses. The amelioration of damage to synaptic morphology and structure may enhance the proximity of receptor channels to the dendritic shaft or enlarge the contact area between neurotransmitters and the postsynaptic membrane, thereby improving synaptic transmission efficacy [32]. PSD-95 is the most abundant scaffolding protein in the excitatory PSD and plays a prominent role in synaptic plasticity. A prior study showed that PSD-95 expression is decreased by soluble A*β* oligomers in AD model mice, concomitant with the downregulation of BDNF/TrkB/CREB signaling [21]. In the current study, we also showed that PSD-95 expression was significantly reduced in A*β*-induced AD rats concomitant with decreased PSD thickness and increased memory deficits. However, this loss was attenuated by ICA treatment, which suggests that an association exists between synaptic protein levels and cognitive function and that ICA modulates cognitive function by altering synaptic plasticity.

A number of reports have shown that BDNF exerts prosurvival effects by binding to TrkB, thereby playing a key role in regulating not only neuronal development, maintenance, and survival but also cognition, memory formation, and memory storage [33]. BDNF-knockout animals show deficits in learning and memory accompanied by reduced BDNF protein expression in the hippocampus [34, 35]. In addition, a reduction in BDNF expression has been found in AD patients [36, 37]. TrkB expression has been shown to decrease in the hippocampal dentate gyrus of transgenic AD mice, and activation of TrkB was shown to protect neurons against A*β*-induced toxicity [38]. BDNF and TrkB also play a critical role in long-term synaptic plasticity [39], and synaptic loss and uncoordinated hippocampal structures have been suggested as important regulators of the various phases of synaptic development [40]. Previous work reported that ICA treatment elevated the mRNA and protein expression of BDNF and TrkB in the hippocampi of AD [41] and depression [42] model rats. The current study confirmed that ICA activates the BDNF/TrkB pathway in the hippocampi of A*β*-induced AD rats, which might contribute to the neuroprotective effects of ICA.

The activation of BDNF engages the Akt signaling cascade in neurons, which promotes the phosphorylation of CREB, a key element in many signaling pathways mediating increased synaptic activity [43, 44]. Several studies have proposed that Akt is involved in A*β*-mediated toxicity in both animal and cellular models of AD. Some studies have indicated that Akt activity is decreased in AD; for instance, Akt activity is decreased in transgenic animal and cellular models of AD [45–47]. However, other studies have reported that pAkt expression was upregulated in extracts of the frontal cortices of AD patients and was positively related to the severity of the pathology [48]. Morroni et al. showed that A*β*_1-42_-induced toxicity corresponded to increased activation of Akt [23]. Our results support this finding, as the pAkt level was decreased in the A*β*-treated model rats. A previous study demonstrated that ICA can regulate Smad-dependent signaling of transforming growth factor-*β*1 (TGF-*β*1) [49], and this neurotrophic factor is known to be a protective and strong activator of the Akt pathway [50]. Our results also show that ICA treatment increased pAkt and pCREB expressions, which was accompanied by BDNF/TrkB pathway activation. This activation appears to be of great significance to synaptic function.

Although our results are consistent with other reports, this study had some limitations that should be clarified. Although the A*β*_1–42_-infused rat model of AD has been widely used to investigate synaptic plasticity in AD, further studies of transgenic models of AD are needed to exclude any effects caused by impairments due to mechanical injury in the A*β* rat model used here. Moreover, ICA is an extract of Herba Epimedii, a classical traditional Chinese medicine, and whether other extracts of Herba Epimedii also exert protective functions in AD remains unclear. We also did not delineate the mechanism through which ICA modulates BDNF expression and synaptic activity in AD-affected neurons. Furthermore, due to experimental limitations, we could not identify specific types of synapses in the hippocampus, such as excitatory or inhibitory synapses or glutamatergic or GABAergic synapses, although we did observe that total levels of PSD-95 increased. Furthermore, BDNF expression is influenced by many factors, such as microRNAs, which can regulate BDNF protein levels through direct targeting of 3′UTR of BDNF mRNA [51]. Thus, we hypothesized that ICA-mediated upregulation of BDNF may be related to microRNAs. Therefore, further investigations of the underlying cellular and molecular mechanisms forming the relationships observed here are still needed.

In the present study, we demonstrated that ICA can alter synaptic plasticity and function through the BDNF/TrkB/Akt pathway by increasing the expression of BDNF and the phosphorylation of its receptor TrkB as well as of Akt and CREB. In addition, ICA appears to improve synaptic plasticity to some extent. These results provide evidence that ICA has a remarkable memory-enhancing effect in AD model rats and suggest that further research on the use of ICA to provide neuroprotection against AD pathology is warranted.
